# Relationship between End-of-Life Care Stress, Death Anxiety, and Self-Efficacy of Clinical Nurses in South Korea

**DOI:** 10.3390/ijerph19031082

**Published:** 2022-01-19

**Authors:** Jihee Choi, Minkyung Gu, Sunyoung Oh, Sohyune Sok

**Affiliations:** 1 Department of Nursing, Graduate School, Kyung Hee University, Seoul 02447, Korea; cjihee9090@naver.com (J.C.); powercyte@naver.com (S.O.); 2 Department of Nursing, College of Science and Technology, Daejin University, Pocheon-si 11159, Korea; g-minkyung@hanmail.net; 3 Department of Nursing, College of Nursing Science, Kyung Hee University, Seoul 02447, Korea

**Keywords:** end-of-life care, stress, death anxiety, self-efficacy, nurse

## Abstract

In South Korea, the number of cancer patients continues to rise, indicating that nurses have greater access to end-of-life care in clinical settings. This study examined the relationship between the end-of-life care stress, death anxiety, and self-efficacy of clinical nurses in South Korea. A cross-sectional descriptive design was used. Participants were 124 nurses working in university hospitals. Data included the general characteristics of study participants, end-of-life care stress, death anxiety, and self-efficacy. Data were collected from February to March 2021. This study shows that the degrees of end-of-life care stress and death anxiety of clinical nurses in South Korea were higher than the median values. Married nurses had higher self-efficacy than unmarried, and there was a difference between bedside and administrative nurses’ self-efficacy. Nurses with no experience of end-of-life care nursing education had higher death anxiety than nurses with experience. The higher the end-of-life care stress of nurses, the higher the death anxiety. The study suggests that therapeutic and detailed educational programs to reduce end-of-life care stress and death anxiety of clinical nurses are needed, and experimental research to verify this. The results can contribute to countries as an additional and enriching reference.

## 1. Introduction

In South Korea, the number of cancer deaths was 80,747 in 2018, and it increased to 83,776 in 2020 [1]. The Hospice Care initiative of 2008 created certified cancer care hospitals in South Korea with a steady rise in terminal cancer care as a result [1]. Despite the advances in medical technology, the number of cancer patients continues to increase, thereby indicating that nurses are likely to encounter end-of-life care in clinical settings more often [2]. Cancer is known to cause death following diagnosis and threaten a person’s physical, social, and spiritual well-being. End-of-life is that part of life where a person is living with, and impaired by an eventually fatal condition, even if the prognosis is ambiguous or unknown [3,4]. End-of-life care combines the broad set of health and community services that care for the population at the end of their lives [2,3]. Thematic analysis of care at the end-of-life identified six main topics of interest, namely, uncertainty of treatment for patients at end-of-life, quality of life issues, costs, ethical and social issues, interaction between medical staff and other services, and strategies for out-of-hospital care [3]. Nurses who provide end-of-life care directly face internal problems and experience higher stress as a result of the excessive expectations of patients or caregivers regarding the progress of cancer treatment, repeated ideals, and disappointments in reality [4,5,6]. If the nurses providing end-of-life care do not have enough time to recharge due to the accumulation of physical and psychological stress, their health and well-being in daily life are impaired [4,7,8]. In addition, they experience the burden, frustration, and depression of the patient who is struggling to face the moment of death and the family that has to let them go, as well as experiencing severe fear and anxiety about death to the extent that they avoid the patient’s death [7,8,9,10].

On the other hand, self-efficacy can be cited as a driving force that motivates nurses in clinical practice and can make changes, thereby implying a strong belief in the individual’s ability to perform a role with respect to their intention [11]. Self-efficacy can be defined as a potential force that nurses can utilize in order to overcome stressful situations when performing end-of-life care to patients [12]. It is also an essential factor in increasing individual resources and strengths [12,13]. As an essential requirement for nurses and a means of effectively achieving the goals of the end-of-life care organization, self-efficacy can help the dying patient’s spiritual well-being and the psychological stability of his/her family [14,15,16,17].

The purpose of this study was to examine the relationship between end-of-life care stress, death anxiety, and the self-efficacy of clinical nurses in South Korea. The aims of this study were (1) to identify general characteristics of the study participants; (2) to examine the levels of end-of-life care stress, death anxiety, and self-efficacy of clinical nurses; (3) to examine the differences on end-of-life care stress, death anxiety, and self-efficacy according to the general characteristics of the study participants; and (4) to examine the correlations among the end-of-life care stress, death anxiety, and self-efficacy of clinical nurses.

## 2. Material and Methods

### 2.1. Study Design and Participants

A cross-sectional, descriptive and correlational design was used in this study. An unknown number of potential participants were approached in person with 148 agreeing to participate and completing the questionnaires. Of the 148 participants, 24 gave incomplete answers to the questionnaires and were dropped from analysis leaving a sample of 124 with complete questionnaires. G-Power analysis with an effect size of 0.25, a significance level of 0.05, and a power of 0.80 determined that a minimum of 123 participants were needed and thus analysis was possible with the 124 participants. Inclusion criteria were those who understood the purpose of the study and agreed to participate voluntarily, those who had at least one end-of-life care experience, and those who worked in a ward with more than one year of clinical experience.

### 2.2. Measures

General characteristics of the study participants included age, marital status, education, clinical experience, position, end-of-life care performance satisfaction, and hospice or end-of-life care nursing education experience. This consisted of a total of seven items.

The end-of-life care stress tool was developed by Lee [18]. It consists of a total of 42 items, including negative attitudes toward the end of life of patients and caregivers, difficulties in allocating time for dying patients, burden on end-of-life care, excessive workload, human conflicts with dying patients, lack of expertise and skills, and conflicts over medical limitations. The score ranges from a minimum of 42 to a maximum of 210 on a five-point Likert scale, with higher scores indicating higher end-of-life care stress. In Lee’s study [18], the reliability of the tool was Cronbach’s α = 0.93, and the reliability of this study was Cronbach’s α = 0.94.

Ko, Choi, and Lee [19] used a death anxiety tool that was translated from the Death Anxiety Scale (DAS) developed by Templer [20]. This tool consists of content on fear of death, denial of thoughts of death, perception of short perspective, and fear of death-related events. A five-point Likert scale was used with a total of 15 items. The score ranges from a minimum of 15 to a maximum of 75, with higher scores indicating higher death anxiety. In the study of Ko et al. [19], the reliability of the tool was Cronbach’s α = 0.83, and the reliability of this study was Cronbach’s α = 0.82.

The self-efficacy tool used by Lee [21] was based on the translation of ‘Specific Self-Efficacy’ of long-term care hospital nurses developed by Pfister et al. [22]. This tool consists of a total of 10 items on symptom control of patients and nursing provision, social environment, psychological problem nursing, counseling and nursing for patient’s family, basic nursing provision, and drug education. A four-point Likert scale was used, with scores ranging from a minimum of 10 to a maximum of 40 and higher scores indicating higher self-efficacy. In Lee’s study [21], the reliability of the tool was Cronbach’s α = 0.82, and the reliability of this study was Cronbach’s α = 0.84.

### 2.3. Procedures

This study was approval by the Institutional Research Board (H-2101-180-1193) of S University. Data were collected from February to March 2021. Researchers visited the hospital and approached all nurses in the hospital and explained the purpose and contents of the study to the preliminary study participants. Only when written consent to participate in the study had been obtained were questionnaire responses allowed to proceed. It took at least 20 min to fill out the questionnaire.

### 2.4. Statistical Analysis

Data were analyzed using SPSS Window 25.0 Program (IBM, Armonk, NY, USA). The general characteristics of the study participants were analyzed using descriptive statistics (frequency, percentage). The levels of end-of-life care stress, death anxiety, and self-efficacy were analyzed using descriptive statistics (mean, standard deviation). The independent *t*-test and one-way ANOVA were used to analyze the differences in end-of-life care stress, death anxiety, and self-efficacy according to the subjects’ general characteristics, and the Scheffe post-test was used. Correlations among end-of-life care stress, death anxiety, and self-efficacy were analyzed with Pearson’s correlation coefficient.

### 2.5. Ethical Considerations

After obtaining approval from the Institutional Research Board of S University, the purpose and procedure of this study were explained to the study participants, and after obtaining consent for data collection, it was revealed that it would not be used for any other purpose than the research purpose. In addition, it was explained that the participants’ anonymity and confidentiality were communicated to them, explaining that they can withdraw at any time during the study if they do not wish to.

## 3. Results

### 3.1. General Characteristics of the Study Participants

The demographics in this study revealed that most (68.5%) were aged 25–34, unmarried (64.5%) and university graduates (79.0%). Most had 5 years or less of clinical experience (37.9%) followed by 5 years and more to 10 years and less (34.7%). Most were general nurses (95.2%), had a moderate end-of-life care performance satisfaction (71.0%). In addition, 35.5% of the participants experienced end-of-life care nursing education (Table 1).

### 3.2. Levels of End-of-Life Care Stress, Death Anxiety, and Self-Efficacy of the Study Participants

When examining the study participants’ end-of-life care stress, death anxiety, and self-efficacy, the total score of end-of-life care stress averaged 167.24, which was higher than the median value 126. The average total score for death anxiety was 48.32, which was higher than the median value of 45. Self-efficacy averaged 29.84 points, which was higher than the median value of 25 points (Table 2).

### 3.3. Differences on End-of-Life Care Stress, Death Anxiety, and Self-Efficacy according to the General Characteristics of the Study Participants

In this study, the end-of-life care stress according to the general characteristics of the study participant showed significant difference according to marital status (t = −0.83, *p* = 0.049). Single nurses had relatively more end-of-life care stress than married nurses.

In this study, death anxiety showed significant differences according to education level (F = 6.61, *p* = 0.002) and hospice or end-of-life care nursing education experience (t = −2.52, *p* = 0.013). Death anxiety was higher in nurses who graduated from college or university than in nurses who graduated from graduate school, and nurses with no experience of end-of-life care nursing education had higher death anxiety than nurses with experience.

Self-efficacy in this study showed significant differences based on marital status (t = 2.09, *p* = 0.039) and position (t = −2.46, *p* = 0.038). Married nurses had relatively more self-efficacy than single nurses, and the difference by position showed that the self-efficacy of the head nurse was higher than that of the general nurse (Table 3).

### 3.4. Correlations among End-of-Life Care Stress, Death Anxiety, and Self-Efficacy

End-of-life care stress, death anxiety, and self-efficacy showed a significant positive correlation between end-of-life care stress and death anxiety (γ = 0.42, *p* < 0.001). In other words, it was found that the higher the end-of-life care stress of nurses, the higher the death anxiety (Table 4).

## 4. Discussion

As regards the general characteristics of the study participants in terms of this study’s aims, most of the nurses participating were unmarried, between the ages of 25 and 34, with the majority having less than 5 years of clinical experience. Given that most of the nurses have less than 5 years of clinical experience, the ward providing end-of-life care is an environment in which it is difficult to work for a long time due to high work stress related to emotional labor [8,23,24]. Continuous and effective management is deemed necessary to improve the nursing work environment in order to reduce the work stress of nurses providing end-of-life care and to provide them with psychological stability [25,26,27].

In this study, the end-of-life care stress and death anxiety of nurses were found to be higher than the median values in each. Given the time frame for this study, the authors would expect COVID to have a significant impact on these results. However, this is consistent with the findings of Woo, Kim, and Kim [28] conducted when there was no COVID, who reported high levels of end-of-life care stress and death anxiety. Regardless of COVID, nurses who provide end-of-life care may consume a lot of physical and psychological energy over time in dealing with dying patients and their families who express anxiety and fear [29]. In particular, it suggests that if the administrative work within the hospital itself related to the end of life is delayed, they may also face difficulties due to aggravated stress [27]. Since most of the subjects were nurses with no experience in hospice or end-of-life care education, it is necessary to develop the job conditions required to create a comfortable working environment by reorganizing the education in the hospital organization to reduce death anxiety [30,31], and developing the process of the ward providing end-of-life care.

Unmarried nurses were found to be more likely to show stress due to end of life care in this study. Since this result is contrary to the findings of Woo et al. [28], it is thought that replication studies should be conducted in the future to reconfirm end-of-life care stress according to the general characteristics of nurses while maintaining homogeneity. The authors infer that the exposure to stress is independent of the person but those who are unmarried may have more problems coping. In addition, nurses who graduated from a 4-year university course but had no experience in hospice or end-of-life care education had higher levels of death anxiety. This suggests that efforts should be made to reduce death anxiety through a systematic step-by-step education, to specifically plan the end-of-life care education program in relation to hospice in the nurse job training [32], and to have a program to reinforce the practical competency of end-of-life care. In this study, the marital status of married and the job position of head nurse or higher were the factors that significantly influenced self-efficacy. Although it is difficult to make an accurate comparison due to the lack of results from the previous study, it supports the findings of Kim et al. [8] in that the general nurses who provided end-of-life care in long-term care hospitals complained of helplessness while performing nursing care without improvement, which can negatively affect self-efficacy. There may be differences in the self-efficacy of bedside nurses and administrative nurses.

Finally, there was a positive correlation between end-of-life care stress and death anxiety in nurses in this study, which is consistent with the findings of several previous studies on nurses [2,7,8,9,23,28]. In order to reduce the nurse’s end-of-life care stress and death anxiety, it is necessary to improve the environment in the process of preparing, to implement sufficient rest, and to improve end-of-life care situations. This can enhance the performance of nurses providing end-of-life care. It is also necessary to accurately diagnose problems in nursing work according to the establishment of a systematic and efficient end-of-life care system in order to supplement it in detail [33,34].

In recent years in South Korea, it has been shown that education for end-of-life care is not compulsory in the continuing education of nurses, and no systematic continuing education programs related to end-of-life practices have been developed and implemented [8]. In relation to cancer, the importance of nursing performance based on end-of-life care expertise is emphasized, which directly leads to end-of-life care and can have a significant impact on the quality improvement of nursing care. As a result, a method for increasing the nurses’ productivity in end-of-life care should be sought.

The above results indicate that a therapeutic and detailed educational program to reduce end-of-life care stress and death anxiety of nurses is urgently needed. In order for nurses providing end-of-life care to achieve high-quality nursing performance, it is necessary to actively recommend a program to help them cope with work stress that may occur during end-of-life care within a medical institution. Based on the results of this study, qualitative research is deemed necessary in the future in order to understand and analyze the nurses’ acceptance of end-of-life care and death anxiety from work stress. Experimental research is also needed to develop a psychotherapeutic program for nurses in charge of end-of-life care and to verify it.

Since the participants of this study were clinical nurses at a university hospital, there are limitations in explaining the end-of-life care stress, death anxiety, and self-efficacy of all clinical nurses in South Korea. It is necessary to conduct an expanded study in consideration of the sample of the study subjects in future. In this study, the collected data were based on a self-administered questionnaire survey with many questions. There are limits to how an individual can express their feelings exactly. The strength of this study is the importance of the research problem. This study is relevant to the nursing profession and adds to the body of work looking at end-of-life care stress and death anxiety in clinical nurses.

## 5. Conclusions

In conclusion, the degrees of end-of-life care stress and death anxiety were higher than the median values. Unmarried subjects had higher end-of-life care stress. Those who graduated from a 4-year university course and had no experience in hospice or end-of-life care education had higher death anxiety. Subjects who are married have increased self-efficacy. There might be a difference between bedside and administrative nurses’ self-efficacy. In addition, the subjects’ death anxiety increased in proportion to their end-of-life care stress.

In future studies the correlations found in this study could be used to build a causal model, helping to identify specific strategies targeting specific groups of nurses to lower their end of life care stress and anxiety. Examination of the specific item responses may also identify specific strategies for future interventions to assist nurses at all levels of educational preparation for end-of-life care.

## Figures and Tables

**Table 1:** General characteristics of the study participants.

Characteristics	*n*	%
Age (year)		
<25	4	3.2
25–34	85	68.5
35–44	25	20.2
45≤	10	8.1
Marital status		
Married	44	35.5
Single	80	64.5
Education		
College	7	5.6
University	98	79.0
Graduate school	19	15.4
Clinical experience (year)		
<5	47	37.9
5–10	43	34.7
11–15	18	14.5
16–20	4	3.2
21–25	8	6.5
26≤	4	3.2
Position		
General nurse	118	95.2
Head nurse	6	4.8
End-of-life care performance satisfaction		
Good	4	3.2
Moderate	88	71.0
Bad	32	25.8
Hospice or end-of-life care nursing		
education experience		
Yes	44	35.5
No	80	64.5

**Table 2:** Levels of terminal care stress, anxiety about death, and self-efficacy.

Variables	Range Point (Median)	Total Score Average Mean ± SD	Average Rating Mean ± SD
End-of-life care stress	42–210 (126)	167.24 ± 19.83	3.98 ± 0.47
Death anxiety	15–75 (45)	48.32 ± 6.54	3.22 ± 0.44
Self-efficacy	10–40 (25)	29.84 ± 5.11	2.98 ± 0.51

**Table 3:** Differences of terminal care stress, anxiety about death, and self-efficacy according to general characteristics of the study participants.

Characteristics	End-of-Life Care Stress		Death Anxiety		Self-Efficacy	
	Mean ± SD	t/F (*p*) Scheffe	Mean ± SD	t/F (*p*) Scheffe	Mean ± SD	t/F (*p*) Scheffe
Age (year)						
<25	189.25 ± 11.08	1.83 (0.145)	50.75 ± 2.06	1.73 (0.164)	30.50 ± 6.19	1.34 (0.264)
25–34	166.11 ± 19.92		49.04 ± 5.94		30.06 ± 5.09	
35–44	168.36 ± 19.96		46.56 ± 7.86		28.24 ± 4.42	
45≤	165.30 ± 18.14		45.60 ± 8.06		31.70 ± 6.20	
Marital status						
Married	165.25 ± 24.13	−0.83 (0.049 *)	48.25 ± 7.51	−0.08 (0.935)	31.11 ± 5.04	2.09 (0.039 *)
Single	168.34 ± 17.08		48.35 ± 5.99		29.14 ± 5.05	
Education						
College	172.86 ± 7.60	2.09 (0.128)	48.43 ± 6.97 a	6.61 (0.002 *) a, b > c	28.14 ± 6.98	0.45 (0.639)
University	168.42 ± 20.85		49.23 ± 5.77 b		29.88 ± 5.07	
Graduate school	159.11 ± 15.31		43.53 ± 8.24 c		30.26 ± 4.75	
Clinical experience (year)						
<5	171.34 ± 18.91	1.79 (0.120)	49.38 ± 5.97	1.90 (0.099)	30.09 ± 5.23	0.86 (0.513)
5–10	162.02 ± 21.04		48.37 ± 5.83		29.54 ± 5.14	
11–15	166.06 ± 19.98		48.33 ± 7.03		28.78 ± 4.01	
16–20	184.00 ± 19.47		39.50 ± 7.14		31.75 ± 7.63	
21–25	169.50 ± 15.03		47.13 ± 10.52		29.38 ± 6.16	
26≤	159.25 ± 11.53		46.25 ± 3.40		34.00 ± 2.94	
Position						
General nurse	167.25 ± 19.74	0.03 (0.976)	48.64 ± 6.38	2.38 (0.059)	29.72 ± 5.19	−2.46 (0.038 *)
Head nurse	167.00 ± 23.51		41.83 ± 6.85		32.17 ± 2.14	
End-of-life care performance satisfaction						
Good	171.75 ± 15.90	2.04 (0.135)	47.50 ± 6.45	0.46 (0.634)	32.25 ± 6.08	0.46 (0.634)
Moderate	164.96 ± 19.61		47.94 ± 6.95		29.77 ± 4.72	
Bad	172.94 ± 20.12		49.44 ± 5.31		29.72 ± 6.07	
Hospice or end-of-life care nursing education experience						
Yes	164.05 ± 18.21	−1.34 (0.184)	46.36 ± 6.54	−2.52 (0.013 *)	29.61 ± 5.82	−0.36 (0.718)
No	169.00 ± 20.56		49.39 ± 6.33		29.96 ± 4.72	

* *p* < 0.05.

**Table 4:** Correlations of terminal care stress, anxiety about death, and self-efficacy.

Variables	End-of-Life Care Stress	Death Anxiety	Self-Efficacy
	γ (*p*)		
End-of-Life Care Stress	1		
Death anxiety	0.42 (<0.001 *)	1	
Self-efficacy	0.14 (0.136)	0.01 (0.946)	1

* *p* < 0.05.

## Data Availability

No new data were created or analyzed in this study. Data sharing is not applicable to this article.
